# Factors Associated with Excessive Body Fat in Men and Women: Cross-Sectional Data from Black South Africans Living in a Rural Community and an Urban Township

**DOI:** 10.1371/journal.pone.0140153

**Published:** 2015-10-08

**Authors:** Kufre Joseph Okop, Naomi Levitt, Thandi Puoane

**Affiliations:** 1 School of Public Health, University of the Western Cape, Bellville, South Africa; 2 Chronic Disease Initiative for Africa, Department of Medicine, University of Cape Town, Cape Town, South Africa; 3 Division of Endocrinology and Diabetes, Department of Medicine, University of Cape Town, Cape Town, South Africa; Hunter College, UNITED STATES

## Abstract

**Objective:**

To determine the factors associated with excessive body fat among black African men and women living in rural and urban communities of South Africa.

**Methods:**

This is a cross-sectional analysis of data from the Prospective Urban and Rural Epidemiology (PURE) study, Cape Town, South Africa conducted in 2009/2010. The study sample included 1220 participants (77.2% women) aged 35–70 years, for whom anthropometric measurements were obtained and risk factors documented through face-to-face interviews using validated international PURE study protocols. Sex-specific logistic regression models were used to evaluate socio-demographic, lifestyle and psychological factors associated with three excessive body fat indicators, namely body mass index (BMI), waist circumference (WC) and body fat percent (BF%).

**Results:**

The prevalence of excessive body fat based on BF%, WC and BMI cut-offs were 96.0%, 86.1%, and 81.6% for women respectively, and 62.2%, 25.9%, and 36.0% for men respectively. The significant odds of excessive body fat among the currently married compared to unmarried were 4.1 (95% CI: 1.3–12.5) for BF% and 1.9 (95% CI: 1.3–2.9) for BMI among women; and 4.9 (95% CI: 2.6–9.6), 3.2 (95% CI: 1.6–6.4) and 3.6 (95% CI: 1.9–6.8) for BF%, WC and BMI respectively among men. Age ≤50 years (compared to age >50 years) was inversely associated with excessive BF% in men and women, and less-than-a-college education was inversely associated with excessive BMI and WC in men. Tobacco smoking was inversely associated with all three excessive adiposity indicators in women but not in men. Unemployment, depression, and stress did not predict excessive body fat in men or women.

**Conclusion:**

The sex-differences in the socio-demographic and lifestyle factors associated with the high levels of excessive body fat in urban and rural women and men should be considered in packaging interventions to reduce obesity in these communities.

## Introduction

Obesity has become a global epidemic with an estimated 1.3 billion overweight or obese adults by 2030[1]; and is a leading preventable cause of death worldwide[2]. Obesity is prevalent in most low- and middle-income (LMIC) countries under transition and is associated with increasing cardiovascular disease (CVD) risk and related-health complications[3–5]. In South Africa (SA), the prevalence of overweight and obesity (referred to as excessive body fat in this paper) has increased steadily over time, reaching 56% in 2002[6] and 65% in 2012[7]; with black African women living in urban townships and some rural communities the most affected. Excess body weight was the cause of 78% of type 2 diabetes, 68% of hypertensive disease, 45% of ischaemic stroke, and 38% of ischaemic heart disease cases among adults in SA [3]. Information on the factors associated with excessive body fat in black African women and men living in rural and urban communities are of critical importance for the development of community-specific obesity prevention strategies in these settings.

The South African black population is experiencing adverse challenges of urbanization and nutrition transitions[8–10]. In SA, obesity is driven mainly by socio-economic and socio-cultural factors including childhood and adult poverty [9,11,12], attitudes about obesity[13], as well as dietary and physical activity behaviours and genetic susceptibility[14,15]. The high rate of overweight and obesity is likely to be sustained in this population due to changing lifestyles[10,14], changing food environment[15–17] and inherent cultural perceptions of body image[13,17] especially among women.

In most regions of the world (including sub-Saharan Africa), recent data indicated high prevalence of excess body weight, with obesity (BMI≥30 Kg/m^2^) far higher than overweight among adults, when compared with earlier reports[1,5,18]. Similarly, the recent South African National Health and Nutrition Examination Survey[7] indicated a substantial variability in overweight and obesity prevalence among adults population based on sex and rural-urban location. For example, overweight/obesity among individuals aged 15 years and above was highest in urban formal (36.0% males and 66.4% females) compared to rural formal locality (23.5% males and 62.3% females). Higher trends of obesity were also reported in urban informal areas compared to the rural informal areas, among boys and girls[7]. The sex differences in the burden of overweight or obesity in rural and urban communities may be due to setting-specific factors that may be sex-specific.

While obesity remains a significant public health concern in South Africa, there is however little or no evidence of innovative preventive strategies, diagnosis, and/or treatment in the resource-poor urban and rural communities. This might be due partly to the lack of appropriate community-level sex-specific information that could support the development of cost-effective setting-specific prevention strategies. Also, in South Africa, no study has assessed the sex-specific factors associated with three measures of excessive adiposity viz. BF%, WC and BMI in black African men and women. Ascertaining sex-specific factors associated with excess body fat based on different adiposity indicators among adults living in rural and urban communities of South Africa is of importance, as a combination of standard body weight measures is recommended for objective assessments of obesity and health risks associated with obesity[19–21].

Previous studies reporting on determinants of obesity had focused mainly on physical activity, diet and socioeconomic factors[11–13,15,18]. Many of these studies were conducted in one specific setting, and usually used body mass index (BMI) and or waist circumference (WC) to assess obesity and health risk, and excluded body fat percent (BF%) measure–which estimates actual body fat more accurately. Moreover, very limited data on BF% obesity among adults in South African communities is however available. This study focused on BF% and WC as indicators which complement BMI as proxies for assessing obesity and health risks[21,22]. Although BMI is the established clinical measurement to estimate CVD risk associated with excess bodyweight, evidence showed that BF% and WC represent better indicators of metabolic[20] and CVD risks[19,21,23] than BMI. A previous study involving white and African American adults also reported that WC and BF% were significantly associated with metabolic syndrome[24]. Moreover, it is commonly reported that BMI cut-off values often used to diagnose obesity have high specificity but low sensitivity to identify adiposity; and do not identify half of the people with high BF%[25,26]. Data from large cross-sectional studies involving Africans indicated that BMI compared to WC and BF% under-estimates obesity particularly in overweight individuals[21,26], and therefore misses those with high risk of cardiometabolic risk factors related to elevated central adiposity.

Body fat percent (BF%) is the proportion of body fat per amount of weight. It is an established standard measure for actual body fat, and is used for health risk appraisal[19,20]. A study by Phillips and her colleagues in 2013 had reported that men and women classified as obese by BF% displayed significant cardiometabolic risks (insulin resistance, pro-inflammatory, pro-thrombotic, and proatherogenic profile) than those classified as obese by BMI[20]. On the other hand, waist circumference (WC) has been traditionally used as a measure of central obesity[19–21]. WC correlates closely with waist/hip ratio and with BMI[27]. Also, an earlier study conducted among South Africans[28], reported that WC was associated with metabolic syndrome (i.e. elevated fasting insulin, triglyceride levels, and low HDL-C levels) in urban black hypertensive women than BMI. The aim of this study was to ascertain the socio-demographic, psychological factors and lifestyle behaviours that are associated with abdominal (WC-defined) and actual body fat (BF%-defined) and general (BMI-defined) excessive body fat among men and women in a rural and an urban South African communities.

In this study, we addressed the following research questions: 1) What proportions of women and men in the rural and urban communities have excessive body fat based on BF%, WC and BMI?; and 2) Does socio-demographic, psychological factors and lifestyle behaviours predict specific excessive body fat indicators differently in men and women living in the rural and urban communities?

## Materials and Methods

### Study setting, population and design

This is a cross-sectional analysis of data of 1220 individuals participating in the Prospective Urban and Rural Epidemiology (PURE) study, Cape Town study center, South Africa. The PURE study is a longitudinal multi-country investigation of the relative contributions of societal influences such as urbanization, nutrition, built environment and lifestyle behaviours on obesity and chronic health conditions including heart disease, diabetes and cancer[29].Two communities are involved in the study; Langa, an urban location (referred to as ‘township’) near Cape Town metropolis, and Mount Frere, a typical rural community located in the Eastern Cape. These are typical resource-poor black-dominated African communities located within different socio-economic environments in transition and with substantial obesity-burden[7,11].

Langa has an estimated population of 52,401 of which 99.1% are black African. The township has grown due to migration of people mostly from the rural Eastern Cape. Like any other black African townships in Cape Town, most residents in Langa live with an average monthly household income of R2,144 ($200) and over 40% are unemployed[30]. The Langa community is grouped into three development areas–“old Langa”, “the Zones” and “the hostels” which mirror the socioeconomic status (SES) of the residents (old Langa is considered higher SES and better established with amenities and the hostels represent the lowest SES). On the other hand, Mount Frere is a rural community located in Alfred Nzo district in the Eastern Cape with an estimated 99.8% black African and an estimate population density of 519 km^2^. Most residents earn an average monthly income between R1001-2500 ($80-$200); with an estimated unemployment rate of over 76%[30].

### Sampling procedure

The two communities were purposively selected based on the feasibility of follow-up for a prospective cohort study and for the purpose of urban-rural comparisons. The methods employed for PURE study have been described elsewhere[29]. For the urban township, systematic sampling was used to select every second household in the three designated development areas. In Mount Frere (rural community), cluster sampling of houses in selected villages under the clan heads was undertaken, since there were no designated street names. From the selected households in both communities, eligible member(s) were recruited as participants for the cohort study. This paper is restricted to 1220 participants aged 35–70 years who had fully completed required physical measurements. This sample represents 60% of the baseline cohort population. Our preliminary analyses showed that there were virtually no differences in the distribution of participants’ characteristics (age, sex, education level, location, and smoking status) between the study sample used for this paper, and the rest of the cohort sub-sample we excluded due to missing physical measurements.

### Data collection

Face-to-face interviews were undertaken using validated international PURE study baseline questionnaires. These questionnaires which were first pre-tested in two South African communities were adapted and used to collect information on participants’ socio-demographic characteristics, medical history and CVD risk factors between March 2009 and June 2010. Information on the risk factors of interest namely stress, depression, physical activity, tobacco and alcohol use, and history of hypertension were also collected.

Exposure variables considered for this study and for which data were collected included i) socio-demographic variables (age, education, location, and employment status), iii) self-reported lifestyle factors (physical activity, tobacco smoking and alcohol use), and psychological factors viz. stress (i.e. feeling irritable, or filled with anxiety at home or at work), and depression (feeling depressed) in the previous 12 months. Other exposure variables considered were self-reported hypertension and unavailability of food at home at any time in the three months preceding data collection. In addition, systolic and diastolic blood pressure (BP) measurements were taken on the left arm of a seated participant using a validated Omron automatic measuring device. An average of two measurements constituted the actual BP. Hypertension was defined as taking BP lowering medication and/or BP ≥ 90/140 mm Hg.

#### Anthropometric and body composition outcomes

Excessive body fat based on the three adiposity indicators (BF%, WC and BMI) were the dependent variables.

Anthropometric measurements were taken using a standard protocol for the study[29]. Height was measured to the nearest 0.1 cm using a standard height meter, and weight was measured to the nearest 0.1kg using a digital weighing scale with subjects wearing light clothing and without shoes. Body mass index (BMI) was generated by dividing weight (in kg) by height (in square meters). Waist circumference (WC) was measured at the smallest diameter between the costal margin and the superior aspect of the iliac crest using a standard measuring tape. During these measurements, participants stood erect and relaxed, with arms at their side. Gold standard methods for measuring BF% such as under-water weighing and dual energy x-ray absorptiometry (DEXA) are expensive and not always practicable in large field studies or for health risk appraisal. Therefore, proxy measures of body fat percent such as skin fold, sex-specific prediction formula based on BMI, as well as bio-electric impedance (BIA) have been used in studies involving black Africans[19,21]. For this study, body fat percent (BF%) for men and women were computed individually using Deurenberg et al[31] and used prediction formula for adults—BF% = 1.20xBMI+0.23xage-10.8–5.4; where sex is 1 for male and 0 for female. This formula has been validated elsewhere[31] in studies involving Africans[32]. Excessive body fat based on the three adiposity indicators (BF%, WC and BMI) were the dependent variables.

Standard WHO cut-offs for BMI and WC[25,33], as well as BF% values used to depict overweight and obesity in studies involving Africans[32,34] were considered for this study. BMI of 18.0–24.9kg/m^2^, 25.0–29.9kg/m^2^, and ≥30.0 kg/m^2^ depicted normal weight, overweight, and obesity respectively. BF% of 30–35% and > = 36% (for women), and 21–25% and > = 26% (for men) represented overweight and obesity categories respectively[34].The corresponding normal body weight categories were values less than overweight. A waist circumference (WC) >88 cm in women and >102 cm in men indicated central obesity[7,33].

### Data analysis

Frequencies and proportions of socio-demographic variables (SDV), and excessive body fat (EBF) based on BMI, WC and BF% cut-offs were calculated by sex. The differences in EBF and SDV between the men and women were determined using chi-square test at 95% significance level. In addition, the mean (SD) values for age, height, weight, and BMI, WC, and BF% for the men and women were compared using independent t-test after stratifying by location. The proportions of participants with EBF by sex and locations, as well as by age categories were also presented. For further analyses, each dependent variable was re-coded as a dichotomous, with excess body weight (i.e. overweight and obesity category) denoted as ‘1’and normal body weight (the reference category) denoted as ‘0’. First, sex-specific bivariate analyses were undertaken to determine the variables that were significantly associated with each of the EBF indicators (unadjusted). The odds ratios (OR), confidence intervals and p-values for the independent variables were determined. The variables that showed significant level of association (p<0.05) with any of the three EBF indicators in the bivariate analyses (viz. location, age, sex, education, employment, tobacco and alcohol use, and hypertension status) and those reported in previous studies[11,12,18] to have significant associations with overweight and obesity (such as physical activity, stress, depression, hypertension, and food unavailability) were considered for multivariate regression analyses. Multivariate logistic regression models were then fitted using a hierarchical (block-wise entry) method to determine the association between independent variables and each of the three EBF indicators, taking men and women as separate cases. The regression analyses produced adjusted odd ratio for each of the independent variables in the models, in relation to the three dependent variables (viz. excessive BMI, WC and BF%). Unemployment and physical activity did not add any significant value to the regression model, as observed in the model fitting statistics (high log-likelihood values) each time they were introduced in the model, and were therefore omitted in the final model. A p-value of <0.05 represented statistical significance. Data was analyzed with SPSS version 22 (IBM Corporation, 2013).

### Ethics Statement

The study and the consent procedures were approved by the Research Ethics Committee of the University of the Western Cape. Permission to work with the baseline data was obtained from the working committee of PURE study Cape Town Centre, and the Population Health Research Institute of McMaster University, Canada—the headquarters of global PURE study. In addition, the study was properly explained to participants with the aid of an information sheet written in participants’ local dialect. All participants provided written informed consent by signing a consent form, after accepting verbally to participate in the study. Participants were duly informed that participation in the study was voluntary and one could opt out at any point. Information obtained during the study was kept confidential. No expected harm was implied to the study participants.

## Results

### Participants’ characteristics and obesity prevalence

The socio-demographic and anthropometric characteristics of the study participants by sex are presented in Table 1. There were 278 (22.8%) men and 942 (77.2%) women; 581 (62%) of the women and 159 (57%) of the men were from the rural community respectively. Mean (SD) age of participants was 50 (10) years; 54.5% of the women and 57.2% of the men were aged between 50 and 70 years. The majority of the participants (82%) were unemployed. About 90% of the men and women had either primary or secondary/high school education, and only 3% had tertiary education. Nearly 37% of women and 46% of men were currently married and about one-third of them were never married. Overall,88.3%, 72.3% and 71.2% respectively had excessive body fat (EBF) based on BF%, WC and BMI cut-offs. Women compared to men had significantly higher prevalence of EBF. The prevalence of excessive body fat (overweight and obesity) based on BF%, WC and BMI cut-offs were 96.0%, 86.1%, and 81.6% for women respectively, and 62.2%, 25.9%, and 36.0% for men respectively.

**Table 1:** Socio-demographic and anthropometric characteristics of study participants.

	Overall		Women		Men		p-value^
	N = 1220		N = 942		N = 278		
Variable	n	%	N	%	N	%	
***Location***							
Urban	480	39.3	361	38.3	119	42.8	0.179
Rural	740	60.7	581	61.7	159	57.2	
Total	1220	100.0	942	77.2*	278	22.8*	
***Age (years)***							
35–49	548	44.9	429	45.5	119	42.8	0.526
50–59	409	33.5	308	32.7	101	36.3	
60–70	263	21.6	205	21.8	58	20.9	
***Education***							
None	49	4.0	29	3.1	20	7.2	0.01
Primary	466	38.2	346	36.7	120	43.2	
High School	668	54.8	540	57.3	120	46.0	
College/University	37	3.0	27	2.9	10	3.6	
***Employment status*** **							
No	825	82.3	630	82.1	195	82.6	0.923
Yes	178	17.7	137	17.9	41	17.4	
***Marital Status***							
Never married	441	36.1	347	36.8	94	33.8	0.01
Currently married	475	38.9	346	36.7	129	46.4	
Co-habiting	49	4.0	33	3.5	16	5.8	
Widow/Separated	255	20.9	216	22.9	39	14.0	
***Percentage body fat*** ^**a**^							
Normal	143	11.7	38	4.0	105	37.8	0.0001
Overweight	193	15.8	126	13.4	67	24.1	
Obesity	884	72.5	778	82.6	106	38.1	
***Waist Circumference*** ^**b**^							
Normal	337	27.6	131	13.9	206	74.1	0.001
Overweight	164	13.4	129	13.7	35	12.6	
Obesity	719	58.9	682	72.4	37	13.3	
***Body Mass Index*** ^**c**^							
Underweight	28	2.3	8	0.8	20	7.2	0.001
Normal	323	26.5	165	17.5	158	56.8	
Overweight	278	22.8	228	24.2	50	18.0	
Obesity	591	48.4	541	57.4	50	18.0	

^p-value obtained using chi-square test (at 95% significance level).

*The proportions (of men and women) are based on the overall total, 1220 (denominator). All other proportions are column percentages.

** Missing data (n = 215) were not included

^a & b^Normal, Overweight and Obesity categories are defined by standard WHO cut-offs for BMI and WC[33]

^**c**^Normal, Overweight and Obesity cut-offs for BF% were taken as<30%, 30–35% and > = 36% (for women), and <21%, 21–25% and > = 26% (for men) respectively[34].

Participants’ mean (SD) age, anthropometric and body fat measures by sex and location are presented in Table 2. There were significant differences in height, weight, BMI, WC and BF%, but no significant difference in age between the men and women in both locations. Women in both locations were heavier than men, whereas men were taller. The mean BMI, WC and BF% for women in the rural and urban locations were respectively higher than the standard cut-off for obesity. For the men, mean BF% and BMI were higher than the standard cut-off values for normal body weight.

**Table 2:** Participants’ mean age, anthropometric and body fat measures by sex and location.

	Urban			Rural		
	Men	Women	Total	Men	Women	Total
Number, n	119	361	480	159	581	740
	(mean, SD)			(mean, SD)		
Age (years)^	51.0 (10)	50.0 (10)	49.9 (10)	51.0 (10)	51.0 (10)	51.2 (10)
Height (cm)*	168.5 (7)	157.0 (7)	160.0 (9)	166.9 (7)	156.9 (6)	159.0 (8)
Weight (Kg)*	75.3 (23)	85.7 (24)	83.2 (24)	66.1 (18)	77.5 (21)	75.1 (21)
BMI (Kg/m^2^)*	26.6 (8)	34.7 (9)	32.7 (10)	23.7 (6)	31.4 (8)	29.8 (8)
WC (cm)*	91.0 (15)	100.1 (16)	97.9 (16)	83.8(10)	93.2 (17)	91.1 (16)
BF% (%)*	27.4 (10)	47.6 (11)	42.6 (14)	24.0 (8)	44.1 (10)	39.8 (13)

^No significant difference (p-value >0.05) between men and women in both locations.

*Significant difference (i.e. p-value <0.001) between men and women for this particular variable in both rural and urban locations. P-values were based on independent t-test (95% CI).

**Fig 1:**
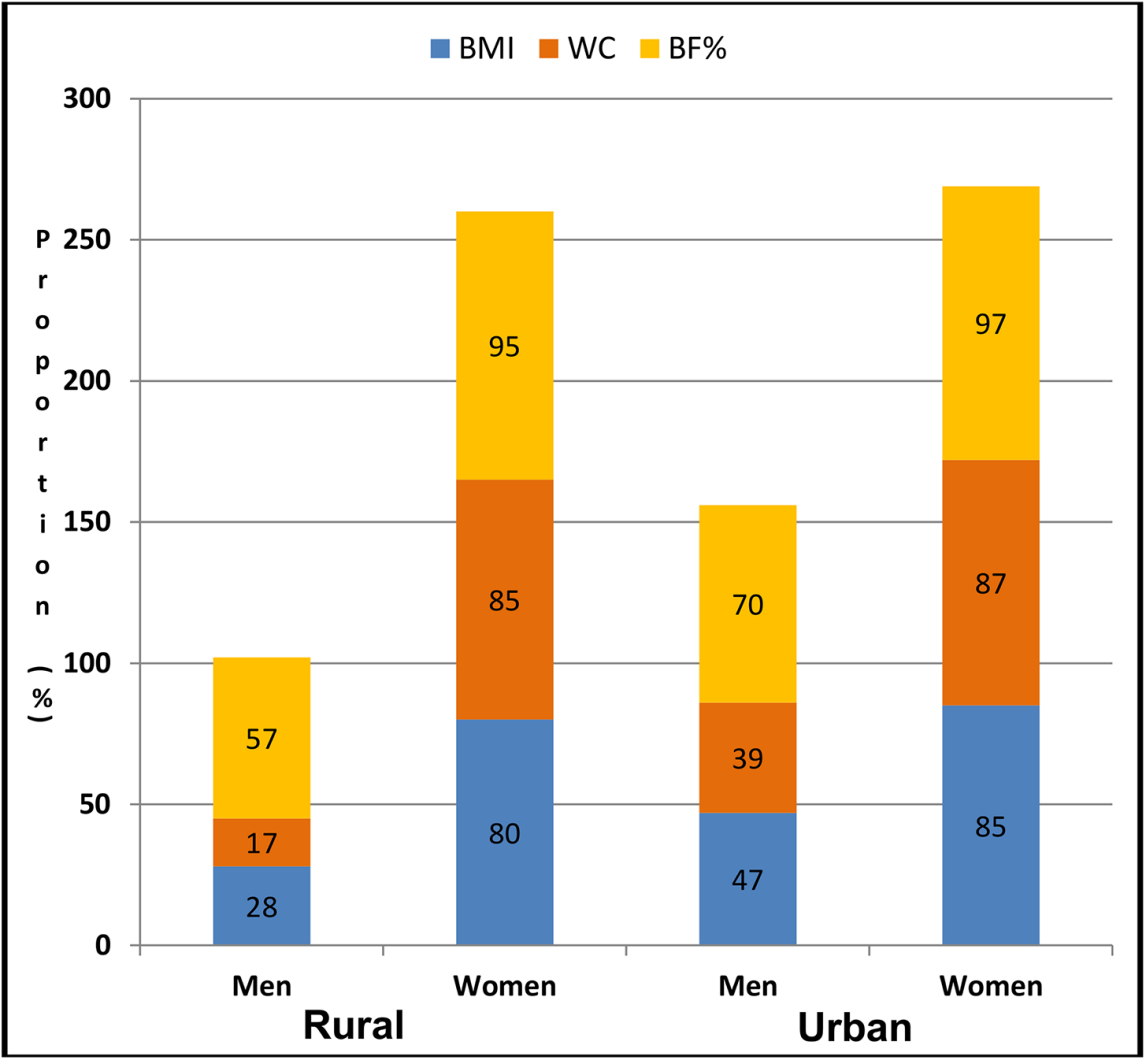
Proportions of men and women with excessive body fat by location. Each block in the bar chart represents a proportion (%) of excessive body fat for men or women (in each location) based on respective adiposity indicator.

In addition, women had higher proportions of excessive body fat than men in both the urban and rural settings for all three adiposity indicators considered (Fig 1). For example, in the rural community, the proportion of women with EBF compared to the men was 95% vs. 57% for BF%, 85% vs.17% for WC, and 80% vs. 28% for BMI respectively.

The proportions of women and men with excessive body fat by location stratified by age are presented in Table 3. Excessive body fat levels increase with age in women and men in both communities, except for WC in the urban women group. For each of the three age categories considered, EBF based on BF% was highest when compared to abdominal and BMI obesity in both the men and women in the two communities.

**Table 3:** Comparison of proportions of women and men with excessive body fat by location and age category.

	Women						Men					
	(N = 942)						(N = 278)					
	Urban (n = 361)			Rural (n = 581)			Urban (n = 119)			Rural (n = 159)		
EBF Proportions	Age Category						Age Category					
	35–49	50–59	≥60	35–49	50–59	≥60	35–49	50–59	≥60	35–49	50–59	≥60
Number (%)*	173 (48)	120 (33)	68 (19)	256 (44)	188 (32)	137 (24)	50 (42)	46 (39)	23 (19)	69 (43)	55 (35)	35 (22)
**EBF (BMI), %**	82.1	86.7	86.8	82.0	72.7	78.8	38.0	45.7	69.6	23.2	25.5	40.0
**EBF (WC), %**	87.9	90.8	86.8	81.3	84.6	90.5	28.0	32.6	69.6	13.0	16.4	25.7
**EBF (BF%), %**	95.4	97.5	100	91.4	97.3	100	54.0	76.1	91.3	42.0	56.4	85.7

* Proportion of women or men are based on the number of men (or women) in each age category in the rural and urban locations.

N/B: Age was significantly associated (p->0.05) with all three EBF indicators in women and men. p-value was obtained by chi-square test.

### Determinants of excessive body fat

The unadjusted odds ratio and 95% confidence intervals (95% CI) for the associations of the socio-demographic psychological and lifestyle variables with EBF is presented in Table 4. Based on the bivariate analyses, current marriage status, location and education showed significant positive association with EBF indicators, whereas lifestyle behaviours namely current alcohol use and tobacco use showed significant inverse association with all three the EBF indicators.

**Table 4:** Bivariate analysis, OR (95% CI) for the association of excess body fat with potential determinants by sex.

	Overweight/Obesity		Abdominal overweight/obesity		High body fat/Obesity	
	(BMI)		(WC)		(BF%)	
Determinants	Women	Men	Women	Men	Women	Men
	OR (95% CI)	OR (95% CI)	OR (95% CI)	OR (95% CI)	OR (95% CI)	OR (95% CI)
**Socio-demographic**						
**Currently married:** Yes	**0.49 (0.34–0.72)** ^**a**^	**3.31 (1.98–5.52)** ^**a**^	0.79 (0.53–1.17)^	**2.84 (1.6–5.0)** ^**a**^	**4.00 (1.55–10.34)** ^**b**^	**4.47 (2.61–7.65)** ^**a**^
(No)	ref	ref	ref	Ref	ref	ref
**Location: Urban**	1.37 (0.97–1.95)^	**2.32 (1.41–3.83)** ^**b**^	1.43 (0.96–2.12)^	**3.01 (1.7–5.2)** ^**a**^	1.55 (0.76–3.17)^	**1.77 (1.07–2.92)** ^**c**^
(Rural)	ref	ref	ref	ref	ref	ref
**Currently employed: No**	0.64 (0.38–1.09)^	**0.47 (0.25–0.92)** ^**c**^	0.76 (0.43–1.36)^	0.65 (0.3–1.3)^	0.60 (0.76–3.17)^	0.68 (0.33–1.40)^
(Yes)	ref	ref	ref	ref	ref	ref
**Education**						
None/Primary	ref	ref	ref	ref	ref	ref
**Secondary-High School**	**1.44 (1.03–2.01)** ^**c**^	**0.10 (0.20–0.48)** ^**b**^	1.03 (0.71–1.50)^	**1.90 (1.1–3.4)** ^**c**^	1.63 90.85–3.15)^	**1.83 (1.10–3.05)** ^**c**^
**College/University**	7.39 (0.99–55.27)^	**0.12 (0.04–0.87)** ^**c**^	4.37 (0.58-32-9)^	**10.24 (2.5–42.2)** ^**b**^	9.10 (3.50–50.01)^	**10.34 (2.10–50.95)** ^**b**^
**Age: >50 years**	0.95 (0.68–1.32)^	1.57 (0.96–2.58)^	1.77 (0.53–1.66)^	**2.24 (1.2–3.4)** ^**b**^	**4.18 (1.90–9.24)** ^**a**^	**3.21 (1.93–5.32)** ^**a**^
(≤50 years)	ref	ref	ref	ref	ref	ref
**Psychological factors**						
**Stress at home** ^**1**^ **: Yes**	1.07 (0.67–1.72)^	1.34 (0.71–2.67)^	1.00 (0.56–1.66)^	0.98 (0.5–2.0)^	0.99 (0.34–2.61)^	1.10 (0.58–2.10)^
(No)	ref	ref	ref	ref	ref	ref
**Depression** ^**2**^ **: Yes**	0.99 (0.71–1.39)^	**0.56 (0.33–0.95)** ^**c**^	0.82 (0.57–1.19)^	**1.84 (1.0–3.4)** ^**c**^	**0.49 (0.25–0.97)** ^**c**^	**0.51 (0.31–0.84)** ^**b**^
(No)	ref	Ref	ref	ref	ref	ref
**Food unavailability** ^**3**^ **: Yes**	1.15 (0.78–1.67)^	0.98 (0.53–1.85)^	0.80 (0.54–1.18)^	0.92 (0.5–1.8)^	1.61 (0.73–3.57)^	1.11 (0.60–2.08)^
(No)	ref	ref	ref	ref		ref
**Lifestyle**						
**Current tobacco smoking** ^**d**^ **: Yes**	**0.47 (0.30–0.74)** ^**b**^	**0.35 (0.21–0.58)** ^**c**^	**0.47 (0.29–0.75)** ^**b**^	**0.39 (0.2–0.70)** ^**b**^	**0.25 (012.-0.51)** ^**a**^	**0.48 (0.29–0.79)** ^**b**^
(No)	ref	ref	ref	ref	ref	ref
**Current alcohol use** ^**d**^ **: Yes**	**0.59 (0.36–0.95)** ^**c**^	**0.28 (0.16–0.49)** ^**a**^	0.69 (0.40–1.19)^	**0.26 (0.1–0.5)** ^**a**^	**0.35 (0.16–0.77)** ^**b**^	**0.46 (0.28–0.75)** ^**b**^
(No)	ref	ref	ref	ref	ref	ref
Physical activity*						
**Low**	1.15 (0.77–1.70)^	1.21 (0.54–2.72)^	0.65 (0.33–1.28)^	1.02 (0.43–2.46)^	0.86 (0.26–2.92)^	1.92 (0.89–4.16)^
**Moderate**	1.23 (0.86–1.75)^	1.62 (0.81–3.21)^	0.59 (0.31–1.10) ^	1.14 (0.54–2.40)^	0.61 (0.21–1.81)^	1.33 (0.70–2.52)^
High	ref	Ref	ref	ref	ref	ref
**Hypertension**						
**Measured or on treatment**						
<90/140 mmHg	ref	ref	ref	ref	ref	ref
≥90/140 mmHg	1.24 (0.97–1.60)^	**0.59 (0.36–0.96)** ^**c**^	1.06 (0.73–1.54)^	**1.95 (1.13–3.36)** ^**c**^	0.86 (0.44–1.67)^	0.70 (0.43–1.14)^
**Measured (only)**						
<90/140 mmHg	ref	ref	ref	ref	ref	ref
≥90/140 mmHg	**1.52 (1.18–1.96)** ^**b**^	**2.00 (1.19–3.24)** ^**b**^	1.40 (0.96–2.01)^	**2.25 (1.30–3.88)** ^**b**^	**2.09 (1.04–4.20)** ^**c**^	2.01 (1.20–3.36)^**b**^

ref = reference category (for respective variables).

^a^p<0.0001

^b^p<0.001

^c^p<0.01

^p>0.05

^1^reported stress at home, and

^2^ any form of depression for at least 3 consecutive weeks in past 12 month

^3^reported staying sometimes without food at home within last 12 month

^**d**^Smoke or consume tobacco products or alcohol at least once a week

*Physical activity based on metabolic equivalent (MET) score/mins: low = MET <600, moderate = MET 600–3000; high = MET >3000).

The multivariate logistic regression analyses that show the factors associated with specific excessive body fat indicators among women and men are presented in Table 5. The results are presented based on the adjusted odds ratio (OR) at 95% confidence interval for each independent variable in the model. The risk (OR, 95% CI) of excessive body fat among the currently married compared to those not married was 4.1 (95% CI: 1.3–12.5) for BF% and 1.9 (95% CI: 1.3–2.9) for BMI for women; and4.9 (95% CI: 2.6–9.6), 3.2 (95% CI: 1.6–6.4) and 3.6 (95% CI: 1.9–6.8) for BF%, WC and BMI respectively for men. Women living in the urban township compared to their rural counterparts were respectively 1.6 times more likely to have excessive BMI or WC, whereas urban men were respectively 2.3 and 3.1 times more likely to have excessive BMI and WC than their rural counterparts. There was however, no significant association between location and excessive BF% for both men and women. Current tobacco smoking was inversely associated with all three indicators of EBF (BF%, WC and BMI) in the women and not in men. In addition, age ≤50 years (compared to age >50 years) was inversely associated with excessive BF% in men and women, and less-than-a-college education was inversely associated with excessive BMI and WC only in men. In contrast, age was not associated with excessive BMI or WC in both men and women. In addition, being hypertensive, or reporting any form of depression or stress had no significant association with any form of EBF neither among women nor men.

**Table 5:** Adjusted odd ratios (95% CI) for association of general, abdominal and percent body fat overweight/obesity with determinants by sex.

	Overweight/Obesity		Abdominal overweight/obesity		High body fat/Obesity	
	(BMI)		(WC)		(BF%)	
Determinants	Women	Men	Women	Men	Women	Men
	AOR* (95% CI)	AOR* (95% CI)	AOR* (95% CI)	AOR* (95% CI)	AOR* (95% CI)	AOR* (95% CI)
**Socio-demographic**						
**Currently married: Yes**	**1.90 (1.26–2.85)** ^**a**^	**3.63 (1.92–6.82)** ^**a**^	1.17 (0.76–1.79)^	**3.18 (1.59–6.39)** ^**b**^	**4.08 (1.33–12.53)** ^**c**^	**4.94 (2.55–9.55)** ^**a**^
(No)	ref	ref	ref	ref	Ref	ref
**Location: Urban**	**1.60 (1.04–2.46)** ^**c**^	**2.30 (1.21–4.51)** ^**b**^	**1.63 (1.01–2.63)** ^**c**^	**3.11 (1.53–6.32)** ^**b**^	2.23 (0.80–6.21)^	1.84 (0.95–3.56)^
(Rural)	ref	ref	ref	ref	ref	ref
**Age: ≤50 years**	1.06 (0.27–1.56)^	0.88 (0.47–1.67)^	0.80 (0.52–1.22)^	0.57 (0.28–1.16)^	**0.21 (0.08–0.52)** ^**b**^	**0.40 (0.21–0.74)** ^**b**^
(>50 years)	ref	ref	ref	ref	ref	ref
**Education**						
None^1^-High school	0.72 (0.49–1.06)^	**0.42 (0.21–0.81)** ^**b**^	1.01 (0.65–1.58)^	**0.36 (0.18–0.74)** ^**b**^	0.45 (0.19–1.07)^	0.89 (0.46–1.70)^
College/University	ref	ref	ref	ref	ref	ref
**Psychological factors**						
**Depression** ^**2**^ **: Yes**	1.19 (0.80–1.70)^	0.69 (0.35–1.36)^	0.93 (0.62–1.42)^	0.73 (0.62–1.42)^	0.60 (0.27–1.35)^	0.67 (0.34–1.29)^
(No)	ref	ref	ref	ref	ref	ref
**Stress: Yes**	0.94 (0.57–1.56)^	1.39 (0.63–3.04)^	0.91 (0.51–1.60)^	0.94 (0.48–2.17)^	0.82 (0.41–1.94)^	1.12 (0.53–2.40)^
(No)	ref	ref	Ref	ref	ref	ref
**Hypertension**						
**Measured or on treatment**						
<90/140 mmHg	ref	ref	ref	ref	ref	ref
≥90/140 mmHg	1.04 (0.72–1.51)^	1.24 (0.68–2.29)^	1.16 (0.77–1.74)^	1.44 (0.74–2.80)^	0.89 (0.10–0.67)^	1.04 (0.56–1.93)^
**Lifestyle**						
**Current tobacco smoking** ^3^: Yes	**0.44 (0.29–0.75)** ^**b**^	0.56 (0.30–1.08)^	**0.51 (0.29–0.93)** ^**c**^	0.63 (0.31–1.29)^	**0.26 (0.10–0.67)** ^**b**^	0.61 (0.32–1.19)^
(No)	ref	ref	ref	ref	ref	ref
**Current alcohol use** ^**3**^ **: Yes**	0.77 (0.40–1.46)^	**0.36 (0.18–0.72)** ^**b**^	0.80 (0.39–1.65)^	**0.31 (0.14–0.67)** ^**b**^	0.44 (0.14–1.44)^	0.77 (0.39–1.56)^
(No)	ref	ref	ref	ref	ref	ref

^*^Adjusted for all other variables in the model; ref = reference category (for respective variables)

^a^ p<0.0001

^**b**^ p<0.001

^**c**^ p<0.01

^p>0.05

^1^Only 4% of the study sample had no education, and where added to any primary & high school education for analyses purpose

^2^Reported being depressed for 2 weeks or more in a row within last 12 months.

^3^Smoke tobacco products or alcohol at least once a week.

## Discussion

This study presented the prevalence of excessive body fat (EBF) based on three standard adiposity indicators, and further determined the socio-demographic, psychological factors and lifestyle behaviours associated with specific EBF indicators among women and men in the study population. Four key findings were demonstrated. First, there were very high proportions of EBF among women (>81%) for all three adiposity indicators. Moreover, six in every ten men (62%) had EBF based on BF%, compared with about three in ten men (36%) with BMI-defined EBF. Secondly, the proportion of men and women with BF%-defined EBF for all age categories were consistently greater than that of WC and BMI. Thirdly, different socio-demographic, lifestyle and psychological factors predicted specific excessive body fat indicators, with distinct differences by sex. Fourthly, less-than- college education in men, and current smoking status among women were inversely associated with at least two of the EBF indicators considered. These findings and their possible explanations and implications for intervention are discussed in the sections below.

### High levels of excessive body fat by sex and location

Excessive body fat in women and men were extremely high, with nine out of 10 women and eight in 10 men with excess BF%. The high proportions of overweight or obesity in our study population is in line with findings of previous studies that reported higher proportions of obesity in women compared to men in South Africa[6,7,12,14] as well as in other developing countries[35]. However, in our study, the level of obesity based on BF%, BMI and WC in men in the communities (rural and urban) was comparatively higher than that reported in other studies in South Africa[6,7,12]. In addition, as age increases, substantially high proportions of men from both the rural community and urban township had excessive body fat for all three adiposity measures considered; with BF% consistently the highest values. These findings suggest that, although obesity is often reported to be higher among South African black women living in urban areas than their rural counterpart[12,14,18], large proportions of both men and women in the rural community are also affected with excess body fat.

In addition, the high proportions of women and men with excessive BF% and abdominal obesity (WC) compared to general (BMI-defined) obesity demonstrates a high burden of obesity and associated health in the two communities. The high proportions of excessive BF% among men (62%) and women (96%) cannot simply be explained by the age-related increase in actual body fat mass and the decrease in fat-free mass with age[36], because BF% overweight/obesity were present in all three age groups considered. These findings are therefore of critical importance, as high BF% is indicative of substantially increased CVD and metabolic risks[37] even in persons with normal BMI[38]. Considering therefore the high cardiovascular risk due to excess body fat, and the prevalence of HIV/AIDS and non-communicable diseases on these communities[14], the implementation of comprehensive prevention interventions that has been advocated overtime[18,39] is urgently and critically needed to reduce the serious health consequences of obesity in these communities. Considering that BMI has been reported as an imprecise measure of visceral body fat and cardiovascular risk[19,20], these findings therefore suggest that using BF% and WC in complement with BMI, should be considered more appropriate for assessing excessive body fat and health risk in this population.

### Socio-demographic factors as predictors of excessive body fat

Our study found that the socio-demographic factors and lifestyle behaviours predicted specific EBF indicators differently in men and in women. For instance, being married, and living in an urban location had a strong positive association with BMI and WC excess adiposity in men and women, but no significant association with BF%. Also, men and women of younger age (<50 years) and men with moderate education were less likely to have excessive body fat for BF%. Similarly, women who currently smoke were less likely to be excessively fat based on all three indicators, whereas in men, no association was found between smoking and EBF. In addition, there was inverse association between alcohol use and excessive body fat for WC and BF% in men. This later finding is in contrast with findings from two prospective cohort studies in the US which reported inverse relationship between alcohol intake and BMI in women, and no relationship in men[40].

The above findings from this study imply that, individual EBF indicators are predicted by different socio-demographic and lifestyle factors, and these factors should be considered in obesity diagnose and CVD risk assessments. This present study collaborated with the report of a recent review which indicated distinct differences between patterns of determinants (i.e. socio-cultural, environmental and behavioural) of obesity in men and women in SA[11]. Specifically, being currently married was significantly associated with all three forms of obesity in men and women, except for abdominal EBF in women. Although reasons for the association between marriage and obesity is not clearly established, marital status has been alleged to alter food consumption behaviours of individuals[13], perhaps due to changing responsibilities and roles after marriage in an African setting. On the other hand, urban location was associated with general and abdominal EBF (not BF%), confirming studies from South Africa[12,14], Nigeria[41] and other LMICs[42]. Similarly, urban location has been found in other studies to be associated with increasing body weight in SA[7,12], and other countries[35,41,42]. The adverse effect of urbanization and globalization[8,16,39] with the resultant adoption of western lifestyles, diet and low physical activity in urban areas largely explains the association between obesity/overweight and urban residence.

Furthermore, there were a number of unexpected findings in this study. The first is the lack of association between education and overweight/obesity among women; whereas in men, less-than-a-college education compared with college education was inversely associated with excessive BMI and WC. Indeed there have been conflicting data regarding the relationship between education and obesity. For instance, an earlier study reported that SA women with no education compared to those with any schooling had higher BMI[6]. Another study reported no association between education and risk of overweight/obesity[12]. Some studies on the other hand had reported association between education and obesity among black South African women[11,18]. The findings from our study, suggest that high education (college or tertiary) status may not have a significant influence on excessive body fat especially among women in these settings.

Secondly, not having food at home (food unavailability) was not associated with EBF for both men and women. Furthermore, the association of obesity and stress and depression had been reported in the United States[43]. The lack of association between stress, depression and food unavailability and EBF might be due to the fact that these variables were determined through self-reported method which perhaps is less objective.

### Lifestyle vs. excessive body weight

Lifestyle behaviours such as physical inactivity has been reported to be responsible for chronic disease and obesity among South Africans[14,15]. However, from our study, physical inactivity was not associated with any EBF indicator neither in men nor women. Similarly, recent studies had also reported lack of association between inactivity and overweight/obesity among South African black adults[12,18]. The lack of association between inactivity and overweight/obesity in the aforementioned studies and this present one is believed to underscore the need for objective instruments to measure physical activity rather than self-reported measures[12]. Tobacco smoking was inversely associated with all three forms of overweight/obesity in women in our study. This finding is of interest even as a recent large cross-sectional study reported a similar finding of an inverse association between smoking and excess body weight among black South Africans adults in Cape Town[18]. However, we need to be cautious when interpreting these results as only cross-sectional data were used. Also, an earlier study in the US had indicated possible association between smoking and weight reduction among adults in some high income countries[44]. The positive effect of nicotine on metabolic rate and its effect on the nervous system levels of norepinephrine, dopamine and/or serotonin, which suppress appetite may explain why smokers tend to weigh less than non-smokers[45].

### Community-based comprehensive obesity control response needed

Public health response to obesity in South Africa and in many LMIC have had very limited success in tackling the rising prevalence of obesity [1,39]. Community-level prevention interventions focusing on children, adolescents and young adults are needed to urgently address the increasing obesity epidemic in order to reduce obesity-related complications and mortality in South Africa. These interventions aimed at reducing obesity and the high non-communicable diseases (NCDs) burden among adults in South African communities should take into consideration the social determinants of health, particularly factors such as age, marital status, community location, and socio-economic, environmental and lifestyle factors. In this regard, the South African Department of Health in its current efforts to reduce obesity and NCDs should implement evidence-based prevention interventions that incorporate appropriate community-level and school-based health education and promotion strategies. Focusing only on physical activity and diets interventions without an understanding of the inherent differences in the determinants of obesity in women and men in the different communities can impede long-term obesity prevention programmes successes.

Lastly, investigating a broader scope of predictors of different forms of obesity by sex and in different settings in SA will inform a comprehensive and effective response to obesity epidemic. In this context, we recommend a robust research initiative that examines individual level and ecological multi-level drivers of excessive body weight, lifestyle and metabolic consequences in order to provide evidence for feasible, acceptable, scalable, and cost effective interventions for obesity prevention. Such initiative should be collaborative and multi-disciplinary, and utilizes the already established population-based cohorts to be able to describe the matrix and dynamics of obesity across communities.

### Study strengths and limitations

Purposive selection of rural and urban locations for the study precluded generalizability of conclusions in the vast resource-poor South Africa. However, these results will serve as important information to support the design and implementation of obesity control strategies in these communities. Earlier studies that examined obesity and its determinants in these communities had excluded BF% which is proven to estimate actual visceral fat. Importantly, the inclusion of body fat percent in assessing obesity in this population is an advantage as it complements WC and BMI measurements. However, BF% values used in this study were estimated using sex-specific equations which have a tendency to over-estimate BF% in obese individuals[32]. Also, the use of self-reported rather than objective methods for measuring physical activity, stress and depression might have limited their accuracy. In addition, the HIV positive status might have resulted also in either weight gain or weight loss in the affected participants[14].

## Conclusion

This study shows a high prevalence of excessive body fat among men and women in the study communities, which is explained by the socio-demographic factors and lifestyle behaviours. The sex-differences in the factors associated with the high levels of excessive body fat in men and women should be considered in packaging interventions to reduce obesity in these communities.
